# Thermoplastic Recycling of WEEE Carcasses with the Incorporation of Talc, Fly Ash, and Elastomers for Composites with Electromagnetic Interference Shielding Characteristics for Electric Car Components

**DOI:** 10.3390/polym17172394

**Published:** 2025-09-02

**Authors:** Mihaela Aradoaei, Alina Ruxandra Caramitu, Magdalena Valentina Lungu, Andrei George Ursan, Romeo Cristian Ciobanu, Magdalena Aflori, Adrian Parfeni

**Affiliations:** 1 Department of Electrical Measurements and Materials, Gheorghe Asachi Technical University, 700050 Iasi, Romania; mihaela.aradoaie@gmail.com (M.A.); andrei_urs@yahoo.com (A.G.U.); adrian.parfeni@student.tuiasi.ro (A.P.); 2 National Institute for Research and Development in Electrical Engineering ICPE—CA, Splaiul Unirii 313, 030138 Bucharest, Romania; magdalena.lungu@icpe-ca.ro; 3 Petru Poni Institute of Macromolecular Chemistry, 41A Aleea Gr. Ghica Voda, 700487 Iasi, Romania; maflori@icmpp.ro

**Keywords:** recycled thermoplastics, WEEE, fly ash, talc, electromagnetic interference shielding, electric car components

## Abstract

In this research, thermoplastic waste (polyethylene and propylene) from waste electrical and electronic equipment (WEEE) was used to manufacture polymer composite materials that included talc, fly ash, and elastomers, with tailored electromagnetic interference shielding properties, for the potential use for electric car components. A distribution of inorganic components within the polymer structures without particle clustering were observed, illustrating an effective melt compounding process. The gradual replacement of talc with fly ash lowered both the fluidity index and the softening temperature values. The increase in fly ash content resulted in higher values of both permittivity and dielectric loss factor. The novelty was related to a significant increase in both dielectric characteristics at increased quantities of fly ash at higher temperatures, an aspect more relevant at higher frequencies where they approached a steady value. The permittivity values surpassed five, and the dielectric loss factor values exceeded 0.04, fulfilling the requirements for their application in electrical equipment. The recipes containing 10% fly ash may guarantee an electromagnetic shielding effectiveness of at least 99% within the frequency domain of 0.1–4 GHz. Composites with greater amounts of fly ash can conduct heat more efficiently, leading to improved diffusivity and thermal conductivity values, with significant thermal conductivity values surpassing 0.2 W/(m*K). Finally, it was concluded that the composites with 10% talc, 10% fly ash, and elastomer using recycled high-density polyethylene might be the best choice for electric vehicle parts, in line with all required standards for these uses.

## 1. Introduction

The European Commission and the Climate Technology Centre and Network (CTCN) report that in the European Union, waste electrical and electronic equipment (WEEE) is increasing each year by 3–5%. Although it seems relatively low, the yearly growth rate indicates that WEEE production is anticipated to double in around 15–20 years, emphasizing the critical demand for efficient management strategies [1]. Improving the collection, treatment, and recycling of WEEE at the end of its life can improve sustainable production, increase resource efficiency, and contribute to the circular economy.

Recovering the components of WEEE is still difficult due to the variety of materials involved [2]. Thermoplastic waste generated from WEEE poses challenges as well as interesting opportunities for recycling [3]. These come mostly from the carcasses of electronic devices and imply distinct regulations for management and disposal because of the existence of harmful substances and possible environmental and health hazards. The recycling technology requires appropriate gathering, disassembly, and material segregation, aiming to reduce environmental effects and enhance resource recovery [4].

In mixed WEEE, 15 distinct varieties of engineering plastics can usually be identified, e.g., as polyethylene (LDPE/HDPE), polypropylene (PP), acrylonitrile–butadiene–styrene (ABS), polyurethane (PU), polyamide (PA), but when referring to selective recycling of carcasses, most predominant are polyethylene (LDPE/HDPE), polypropylene (PP), and acrylonitrile–butadiene–styrene (ABS) [3,4]. In addition, some additives (both organic and inorganic) are already incorporated into plastics to confer specialized features, such as the following: color, impact resistance, flammability, weathering resistance, etc. Such additives may include inorganic oxides (e.g., TiO_2_, ZnO, Cr_2_O_3_, Fe_2_O_3_), flame retardants, stabilizers, and/or plasticizers, according to the applied thermoplastic technology [2]. The implementation of the Restriction of Certain Hazardous Substances (RoHS), according to the Directive 2002/95/EC [5], has constrained the presence of these hazardous substances. However, the WEEE that is currently being processed still contains these additives, affecting the further processing [6,7]. It is considered that innovative research on recycling processes can provide important insights to ensure eco-design measures and to determine the effectiveness of any technological measures aiming to increase the recyclability of products at the design stage [8,9,10,11].

As previously demonstrated in the literature, thermoplastics modified with talc (hydrated magnesium silicate) and conductive additives (especially metallic or carbon) may find applications in various fields, with talc mainly used for improved mechanical properties and conductive fillers for tailoring the electrical conductivity. Thermoplastics filled with talc are used in automotive parts due to their improved rigidity, dimensional stability, and surface finish. Conductive thermoplastics are crucial in electronics for electrostatic dissipation, electromagnetic interference (EMI) shielding, etc.

Studies upon polyethylene composites with talc [12,13,14,15], or polypropylene composites with talc [16,17,18,19] demonstrated an increase in physical and mechanical properties, making talc an effective additive intended for large temperature applications, with reduced shrinkage by promoting uniform formation and enhancing cooling efficiency. On the other hand, talc offers a cost-effective alternative to other fillers and additives commonly used in thermoplastics. By replacing heavier fillers or reinforcing agents with talc, it lowers material density while preserving mechanical strength, stiffness, and heat resistance and assuring a lower thermal expansion. Some combinations of talc and other mineral additives, such as alumina [20,21] or calcium carbonate [22], within thermoplastic composites may enhance their performance. Recent studies have also taken into account the combination of talc and elastomers within thermoplastic composites [23,24,25], in order to improve their characteristics such as flexibility, durability, and resistance to impact. Elastomers generally act as compatibilizers, enhancing the interaction between different phases in the composites and contributing to overall material performance, especially in terms of chemical, water, and UV radiation resistance. In this context, talc/elastomer-filled thermoplastics may be successfully used in automotive applications, where maintaining performance and durability between −50 °C and +90 °C is currently required [26].

Previous studies also analyzed the use of fly ash, a by-product of coal combustion, as an additive in thermoplastic composites in principle as a reinforcing and flame-retardant filler used alone [27,28,29,30,31,32], or in combination with other inorganic additives [33,34,35,36,37], or in combination with elastomers [38]. The main purpose was the enhancing of thermo-mechanical features of composites, mainly for building applications [39], but with the potential of use for automotive applications too [40]. The main gain considered for using fly ash as an additive was related only to sustainability issues and its low cost, and the main reason to test it was to substitute other fillers which are more costly. But no research addressed its potential as an additive in thermoplastic composites for offering shielding against electromagnetic interference due to its composition, including a significant content of Fe/Cr/Ni oxides. The use of fly ash for materials with tailored electromagnetic shielding performance was mentioned in the literature, but exclusively in relation with building materials, either based on cement/geopolymers [41,42] or other types of complex panels [43,44].

Electronic equipment enclosures, also referred to as cases, must fulfill various criteria to guarantee the safety, performance, and durability of the devices they contain, especially when used in the automotive industry. These specifications include protection against physical damage, environmental factors, water and dust resistance, and electromagnetic interference, alongside factors related to optimal dimensions, weight, and installation process. The requirements are even stricter when addressing electric car components, where more sensitive electronics are involved. The case materials technology needs electromagnetic compatibility issues to guarantee that equipment operates correctly without generating or being influenced by electromagnetic interference (restricted electromagnetic emissions and immunity to interference), in this way avoiding operational failures and disruption to other equipment [45]. Consequently, the optimal choice of materials for electric car components must offer a balance of mechanical strength, electrical insulation, thermal management, and resistance to environmental factors like moisture, chemicals, and electromagnetic interference [46].

No study known up to date has tackled the use of fly ash as a component in composites with electromagnetic interference shielding characteristics for automotive applications, especially for electric car components. On the other hand, the use of recycled thermoplastic matrices from WEEE for specialized composites with tailored electrical properties for electronic equipment enclosures was not addressed in the literature.

In our work, we aimed to utilize thermoplastic waste (polyethylene and propylene) from WEEE by creating polymer composite materials with hybrid fillers. The innovation of the study is related to the development and testing of thermoplastic composite materials based on talc, fly ash, and elastomers in order to be potentially used for automotive applications, especially for electric car components, i.e., electronic equipment enclosures with tailored electromagnetic interference shielding characteristics.

## 2. Materials and Methods

### 2.1. Materials

The polymer composite materials were prepared using the following raw materials:

- HDPE, LDPE, and PP recycled from WEEE waste (denoted as HDPEDE, LDPEDE, and PPDE, respectively), technologically obtained by ALL GREEN SRL, Iași, Romania, with the methodology and characteristics being described in [44]. Before use, the recycled polymer matrices were regranulated into pellets with a size of approximately 2 mm.
- Industrial talc (Ta) (Imerys S.A., Paris, France), up to 20% (wt%),
- Thermal power plant ash of Romanian origin (FA), with particles average size under 20 μm and a D50 of 9.1 μm, up to 10% (wt%).
- A 10% (wt%) elastomer type: ethylene-methyl acrylate-glycidyl methacrylate terpolymer, Lotader AX 8840 (SK Functional Polymer S.A.S., Courbevoie, Île-de-France, France).
- Up to 3% (wt%) compatibilizing and coupling agents in all developed composites, including PEGMA 400 (Polysciences, Warrington, PA, USA), an Ethylene Acrylic Acid Copolymer, and Tegomer E 525 (Evonik Operations GmbH, Essen, Germany).

### 2.2. Equipment and Methods

#### 2.2.1. Obtaining the Composite Materials

**Figure 1:**
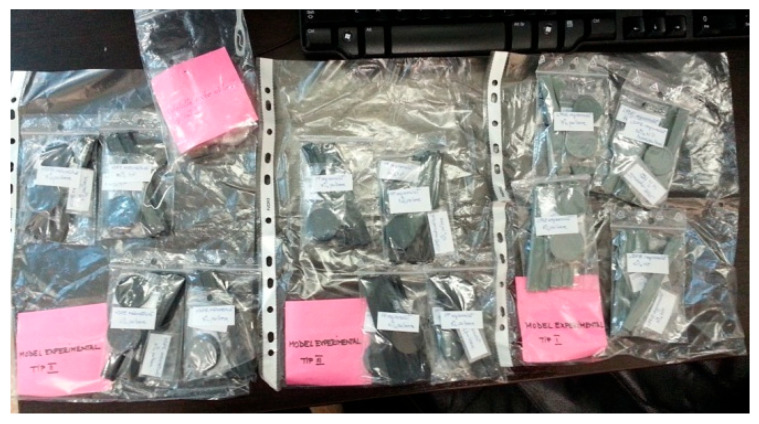
Disk-shaped and parallelepiped samples.

The raw materials, in powdered form, were mixed together for 15 min in a TURBULA T2F cylindrical mixer (Artisan Technology Group, Champaign, IL, USA), with a rotation speed of 40 rpm. The obtained mixtures were subsequently placed into the feed hopper of a Dr. Boy 35A injection machine (Dr. Boy GmbH & Co. KG, Neustadt-Fernthal, Germany), where they underwent an injection molding process, with key operating parameters detailed in [26,44]. The temperatures in the screw zones of the machine were kept between 150 and 190 °C, and the applied pressure varied from 116 to 120 kN, to create disk-shaped and parallelepiped samples with a thickness of 2 ± 0.1 mm, according to the needed sample types for the specific tests, as shown in Figure 1.

**Figure 2:**
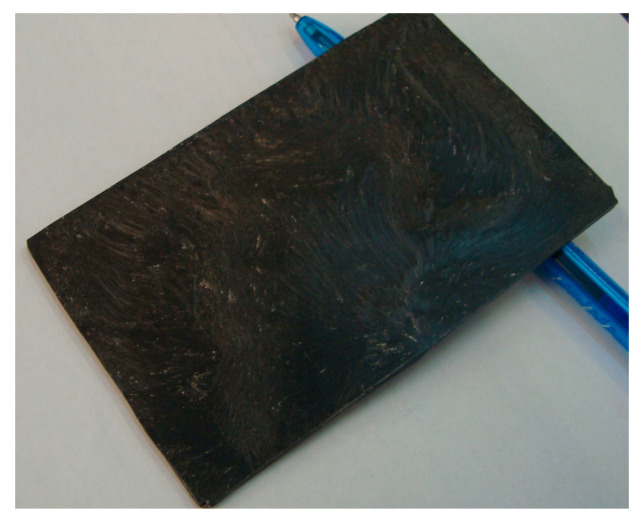
Extruded plate sample.

In the second stage, the obtained mixtures were submitted to an extrusion process for obtaining larger plates with a thickness of 2 ± 0.1 mm, to be embedded within a specialized frame for thermal shielding and electromagnetic compatibility tests, as seen in Figure 2. The equipment used was a laboratory twin-screw extruder, MD 30 (Bausano, Torino, Italy).

#### 2.2.2. Characterization Methods

(i) Electron scanning microscopy SEM was performed with a field emission and focused ion beam scanning electron microscope (SEM), model Quanta FEG 250, with STEM and EDX detectors (Thermo Fisher Scientific Inc., Waltham, MA, USA).

(ii) Elemental chemical analysis by X-ray fluorescence spectrometry (XRF) was performed on the Bruker XRF analyzer S8 TIGER X-ray fluorescence spectrometer (Germany).

(iii) A Netzsch STA PC 409 thermal analyzer (Erich NETZSCH B.V. & Co. Holding KG, Selbwas, Germany) was used for thermogravimetric analysis. The heating program was 35–1200 °C, with a heating speed of 10 °C/min.

(iv) Physical characterization presumed the following procedures:

- Melt flow rate and melt density were determined according to [47] with a Lab BP-8164-B Melt Flow Index Tester (Dongguan Baopin Precision Instrument Co., Ltd., Dongguan City, Guangdong Province, China).
- Ash content of samples was determined using an LVT calcination oven (Nabertherm GmbH, Lilienthal, Germany) and a UF 55 forced convection oven (Memmert GmbH + Co. KG., Schwabach, Germany). The samples were first dried in the oven to remove moisture, then burned in the oven, and the tests were carried out according to [48].
- The VICAT soaking temperature test was carried out according to [49].

(v) The density of materials was determined using a XS204 hydrostatic balance (Mettler-Toledo, Greifensee, Switzerland), according to [50], as an average of 5 specimens per material.

(vi) Shore hardness measurements were taken with a common Microdurometer Vickers FM700 (Future-Tech Corp, Tokyo, Japan), as the mean of 5 measurements, [51].

(vii) The equipment for determining the mechanical features was a specialized PC-controlled universal tensile testing machine (Qiantong, China), with a nominal force of min 20 kN, allowing measurement of tensile strength and elongation, as indicated in [52,53]. Charpy impact strength was tested according to [54] on an Izod/Charpy impact tester (Qualitest, London, UK).

(viii) The dielectric properties were carried out using a broadband dielectric spectrometer (Novocontrol GMBH, Montabaur, Germany) encompassing an alpha frequency response analyzer and a quattro temperature controller, with tailored measurement cells, in the frequency band of 10 Hz–10 GHz.

(ix) The resistivity tests were carried out using a Keithley 6517B/E electrometer (Tektronix/Keithley, Cleveland, OH, USA), in accordance with the procedure described in [55].

The dielectric strength test was performed according to the method outlined in [56], with an applied voltage rate of 2 kV/s.

(x) Electromagnetic compatibility tests were carried out by applying the coaxial transmission line method and measuring the reflection in free space, with coaxial transmission devices, anechoic chamber, and Schwartzbeck antennas (Schönau, Germany). The main equipment used was a signal generator SMCV100B RF (Rohde & Schwarz, Munich, Germany) in the frequency band of 1 kHz–4 GHz, and a signal analyzer FPC1500-Desktop Spectrum Analyser (Rohde & Schwarz, Munich, Germany), in the frequency band of 1 kHz–4 GHz, according to IEEE 299-2006 [57].

(xi) The degree of swelling (water and solvent absorption) was determined by measuring the variation in the mass of the samples at predefined immersion intervals, utilizing the XS204 Analytical Balance (Mettler-Toledo, Greifensee, Switzerland), according to ISO 62:2008 [58], after 168 h of immersion.

(xii) Laser flash analyzer equipment, model LFA457 (NETZSCH-Gerätebau GmbH, Selb, Germany), was used to determine the thermal shielding parameters: 5 determinations were made for each sample.

(xiii) Martens thermal stability is achieved by exposure to progressive temperatures up to 150 °C in a UF 55 forced convection oven (Memmert GmbH + Co. KG., Schwabach, Germany), using samples with standard dimensions (length 120 mm, section 10 × 20 mm^2^), according to [59] and associated with a Vicat test [60].

## 3. Description and Characterization of the Studied Materials

### 3.1. Composition and Labeling of the Studied Materials

The composition and identification of the analyzed materials are shown in Table 1 (M1–M3 with LDPEDE matrix, M4–M6 with HDPEDE matrix, and M7–M9 with PPDE matrix). The melting intervals for the obtained composites (M1–M9) are presented in Table 2.

### 3.2. Characterization of the Studied Materials

#### 3.2.1. SEM Analysis

**Figure 3:**
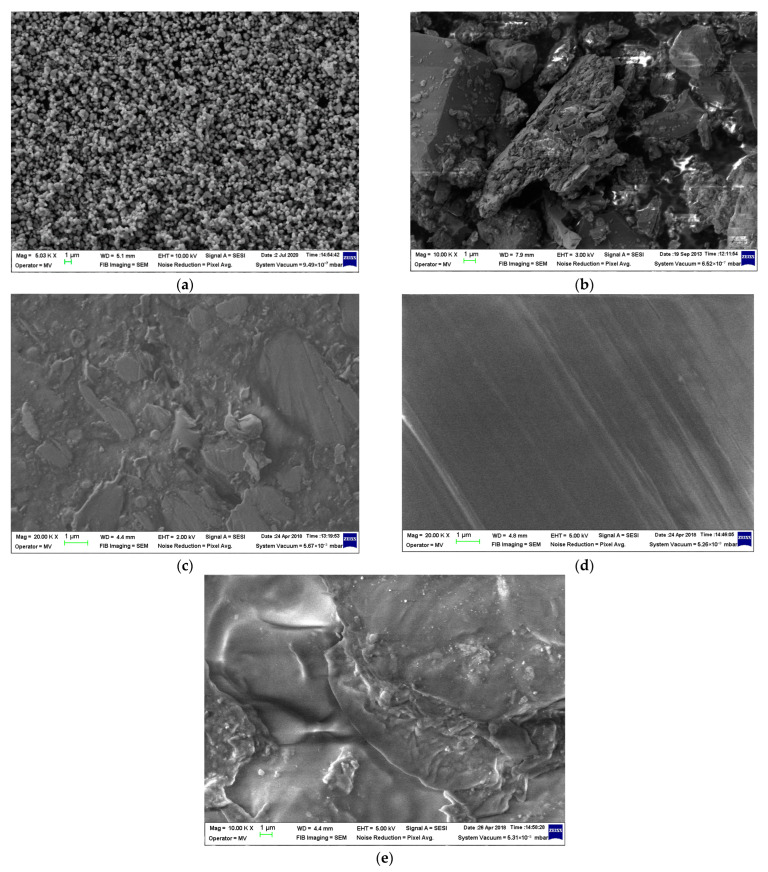
Micrographs for (**a**) talc powder, (**b**) fly ash, (**c**) LDPEDE, (**d**) HDPEDE, and (**e**) PPDE.

The micrographs obtained for the raw materials are presented in Figure 3.

Based on the obtained SEM image, Figure 3a, the talc powder was observed to be relatively uniform in dimension, with an average close to 1 μm. In regard to fly ash, Figure 3b, one can notice a mixture and conglomerate of particles of irregular shapes which may turn friable into smaller dimensional powders during the mixing process.

In regard to the matrix structure, the recycled LDPE and PP seem to mix micro-structures with irregular forms, Figure 3c,e, compared to HDPEDE, Figure 3d, which seems to maintain a more uniform structure, similar to the initial virgin polymer form.

**Figure 4:**
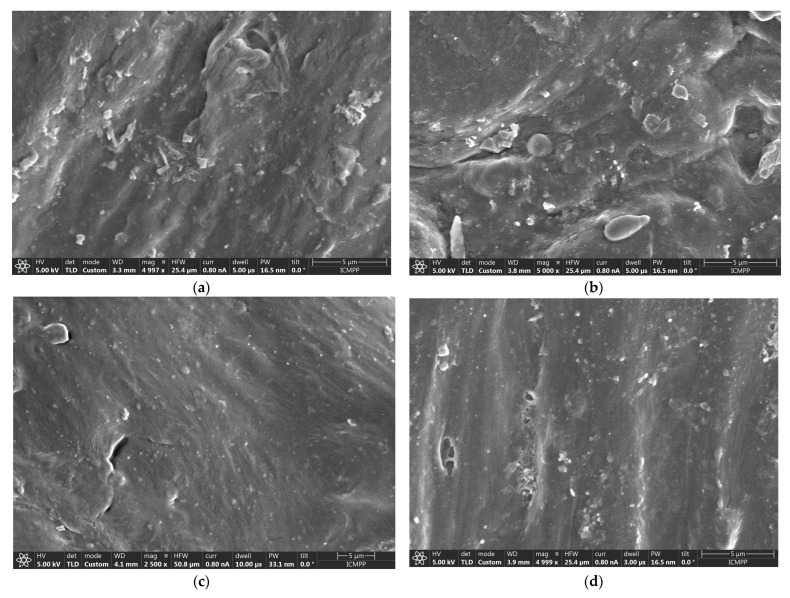

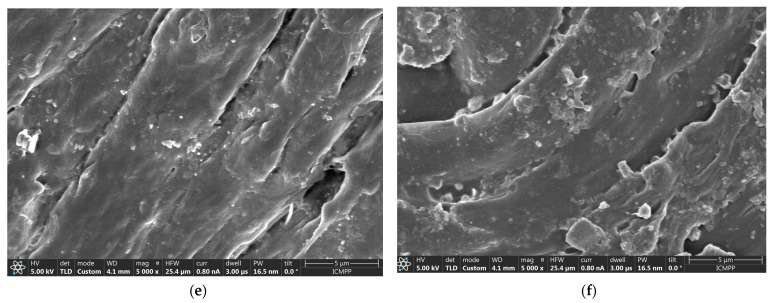
Micrographs for (**a**) M1, (**b**) M3, (**c**) M4, (**d**) M6, (**e**) M7, and (**f**) M9.

In regard to the composites structures, they are observable in Figure 4. In all circumstances, it is obvious that the partial substitution of talc with fly ash is hardly noticeable as the particles distributed among the polymer structures are in quite similar dimensions, meaning that the fly ash was chopped and dispersed into micro-particles comparable in dimension to talc, all being mixed and subjected to a relatively uniform dispersion. When comparing the structures of initial matrices, Figure 3c–e, with the composites containing elastomer, a more uniform and consistent form of matrices was obtained, especially in the case of LDPEDE and PP, as seen in Figure 4a,b and Figure 4e,f, respectively. On the other hand, no significant alteration was noticed in the case of HDPEDE when comparing Figure 3d with Figure 4c,d. In all, that means that the addition of the elastomer enhanced the quality of recycled polyolefins as matrices during the thermoplastic process.

This relatively uniform dispersion with the aid of the elastomer and with no agglomeration of inorganic particles suggests an effective mixing during melt compounding, which may contribute to the overall structural integrity of such composites which are obviously superior to those lacking elastomer [14,18,23,24,25,44].

#### 3.2.2. XRF Analysis of the Fly Ash

**Figure 5:**
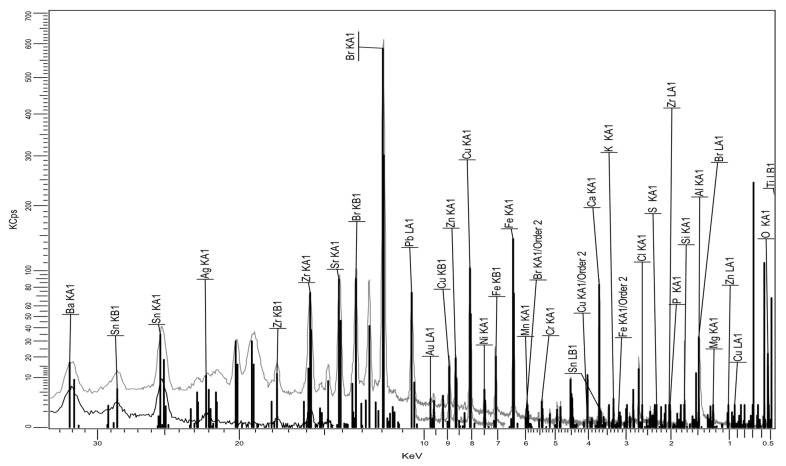
XRF pattern of the fly ash.

The XRF pattern of the fly ash is presented in Figure 5, and the quantitative analysis in Table 3.

It is obvious that the fly ash includes a large variety of metallic oxides, some of them compatible with talc and enhancing the effect of talc in regard mainly to the mechanical properties, as observed also in [20,21,22], and others offering potential ferritic properties of such powders. This is to be innovatively demonstrated further when analyzing the behavior of composites at higher frequencies.

#### 3.2.3. XRF Spectrum for Recycled Matrices

**Figure 6:**
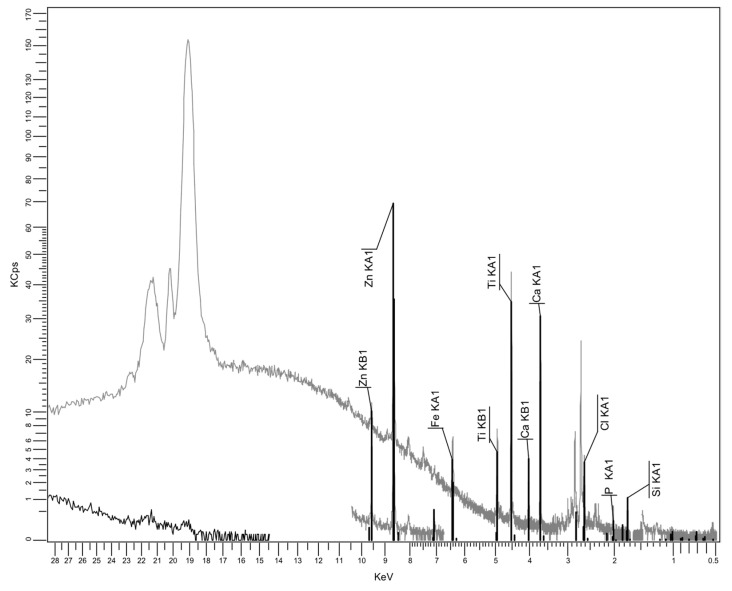
XRF spectrum for LDPEDE.

**Figure 7:**
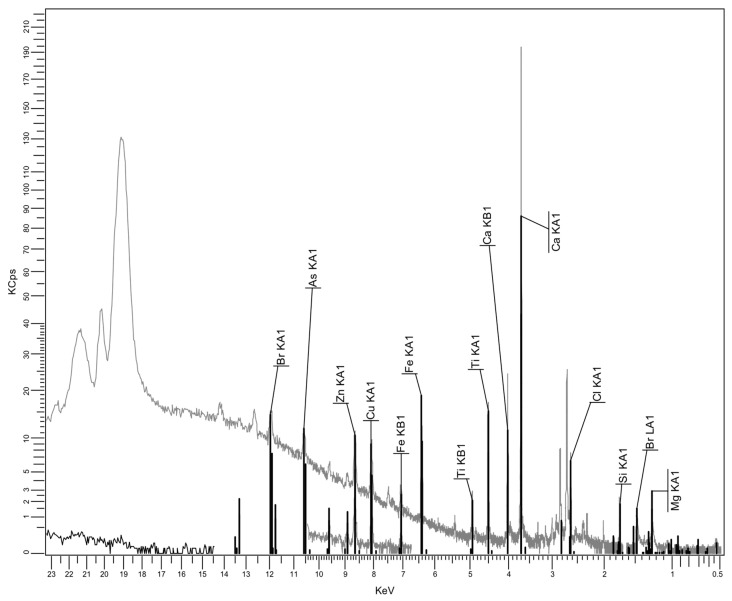
XRF spectrum for HDPEDE.

**Figure 8:**
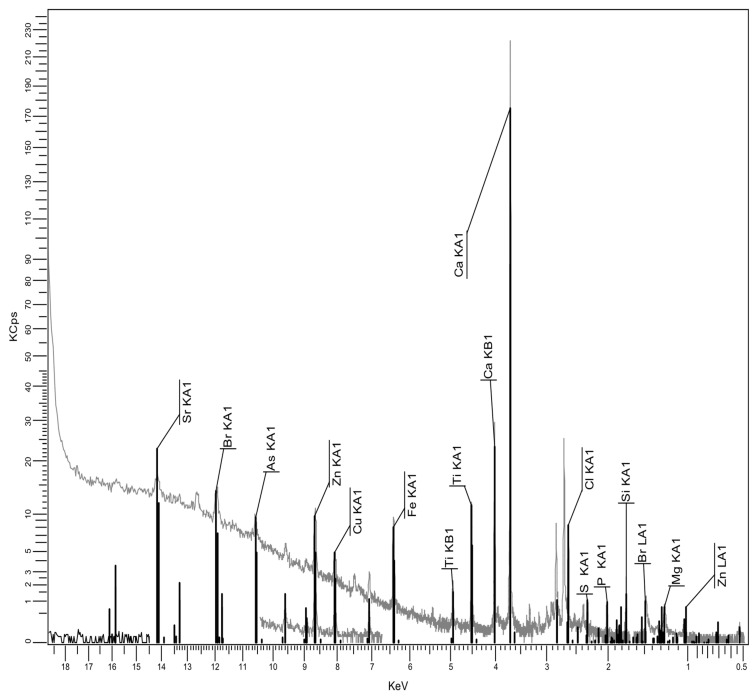
XRF spectrum for PPDE.

An important issue is related to the analysis of the composition of recycled polyolefin matrices from WEEE. This is because they are expected to contain additional substances, some of them introduced for technological purposes while others arose due to contamination effects. The XRF spectrum results are presented in Figure 6, Figure 7 and Figure 8, and the analytical analyses are in Table 4, Table 5 and Table 6.

It seems that PPDE, Table 6, presents the largest variety of additional residual compounds, but the content of most of them may be negligible. On the other hand, the most significant ingredients are, in all cases, CaO, TiO_2_, and SiO_2_, which are particles fully compatible with talc, as mentioned before when discussing the effect of fly ash composition. Finally, the presence of Fe_2_O_3_ in all recycled matrices is beneficial from the electromagnetic point of view. No relevant heavy metal as type or percentage was identified. Consequently, the compositions of recycled matrices and of fly ash offer a good background for obtaining specialized composites for electric car components.

Particular focus should be given to the potential presence of flame retardants within recycled polymer matrices, especially in the case of brominated flame retardants. One can notice the Br occurrence only in relation to HDPEDE and PPDE, with an extremely low concentration. That is because the research took into account only the carcasses, which constitute the most relevant source of recyclable polyolefins, where the flame retardants are not generally used in large quantities. It is obvious that through improper recycling methods like open burning, flame retardants, being persistent and bioaccumulative chemicals, could be released into air, contaminating the environment. However this is not present in our case, which uses controlled thermo-mechanical processing. It is clear that, as demonstrated in the literature, the use of recycled thermoplastics containing flame retardants for similar thermoplastic technologies brings two major advantages: the diminishing of the new addition of flame retardants to meet the technical requirements of the application, and, on the other side, the indirect control of the pre-existing flame retardants which are reincorporated in new components [44] without being eliminated into the environment.

#### 3.2.4. Thermogravimetric Analysis of Recycled Thermoplastic Matrices

**Figure 9:**
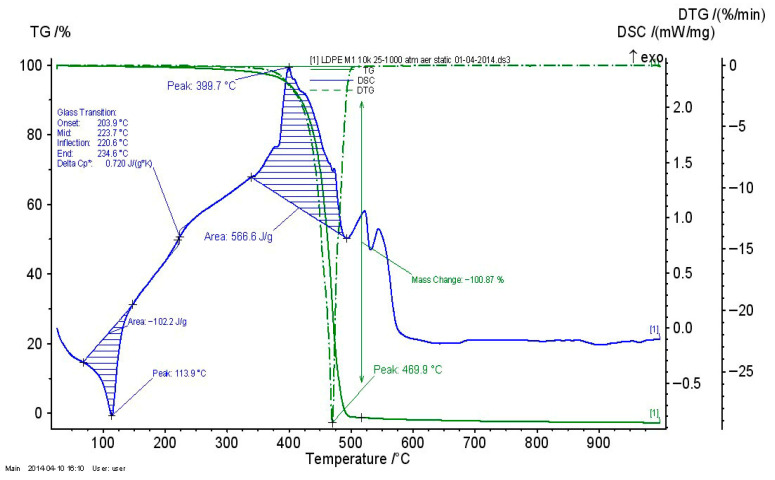
TG_DSC curves for LDPEDE.

**Figure 10:**
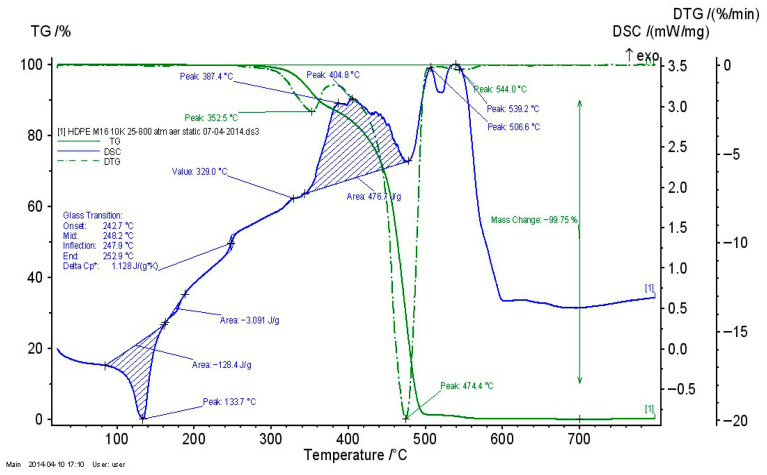
TG_DSC curves for HDPEDE.

**Figure 11:**
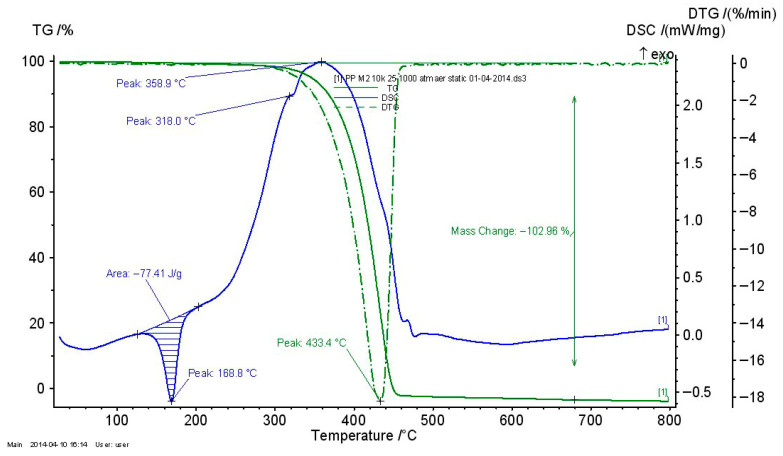
TG_DSC curves for PPDE.

The thermogravimetric analysis of recycled thermoplastic matrices is presented in Figure 9, Figure 10 and Figure 11.

For all recycled matrices, the thermal behavior consisted of a melting process and an oxidation–thermo-oxidation process. The data are presented in Table 7. A vitreous transition in the case of PPDE was not identified.

#### 3.2.5. Physical Analysis

The analysis of physical parameters, Table 8, reveals that even if theoretically the percent of inorganic ingredients in composites stands at 20% (wt%), the gradual replacement of talc with fly ash at 5%, and finally at 10%, diminishes the fluidity index and softening temperature values. An explanation may be related to the homolog diminishing of both bulk and melt density values due to the lower density of fly ash compared to talc powder. This assertion is partially supported by the change in ash content following combustion [48], which also decreased with the progressive substitution of talc with fly ash. In this case, the reduction is not as significant as anticipated since the combustion of talc generates magnesium oxide (MgO) and silicon dioxide (SiO_2_), along with water vapor, which are eliminated. As a result, fly ash remains unchanged under combustion, while talc decomposes and loses mass at high temperatures; nevertheless, the mass reduction associated with talc cannot compensate the density difference when referring to the leftover materials in ash. The conclusions apply to all the polymer matrices involved, although the effective values and decreasing intensities varied.

#### 3.2.6. Electrical, Dielectric, and Electromagnetic Compatibility Tests

**Figure 12:**
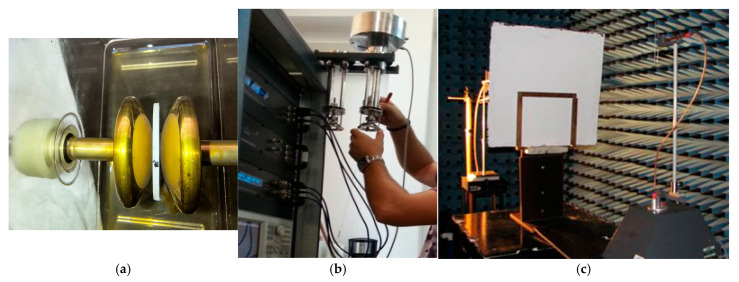
Experimental equipment: (**a**) breaking strength cell; (**b**) broadband dielectric spectroscopy equipment; and (**c**) coaxial transmission line in anechoic chamber.

In Figure 12, test equipment for breaking strength, dielectric spectroscopy and, respectively, electromagnetic shielding are briefly presented. The most important features of the analyzed composites when referring to their potential applications for electric car components are related to dielectric analysis (dielectric permittivity and dielectric loss factor).

**Figure 13:**
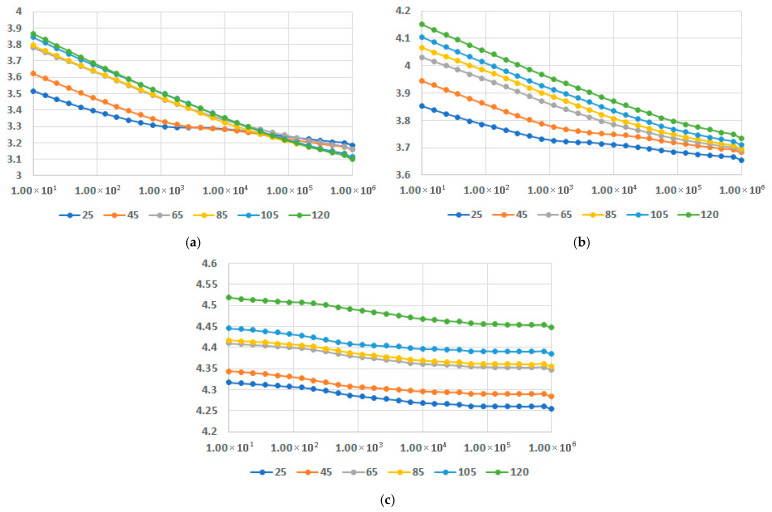
Dielectric permittivity vs. frequency [Hz] at different temperatures [°C] for (**a**) M1, (**b**) M2, and (**c**) M3.

**Figure 14:**
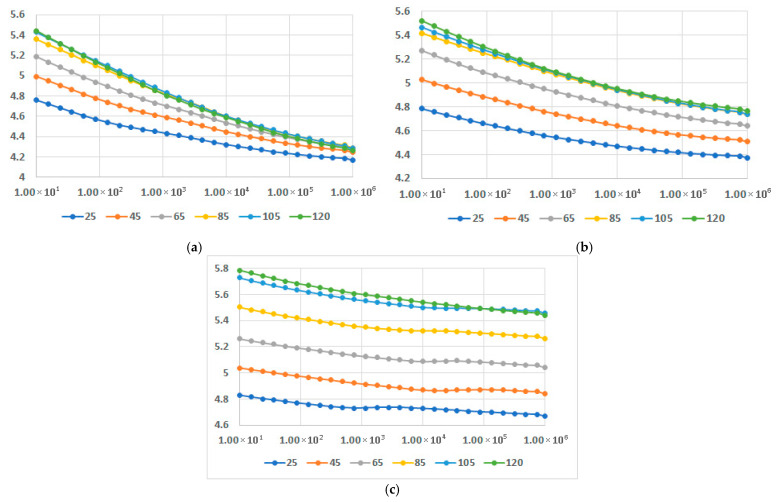
Dielectric permittivity vs. frequency [Hz] at different temperatures [°C] for (**a**) M4, (**b**) M5, and (**c**) M6.

**Figure 15:**
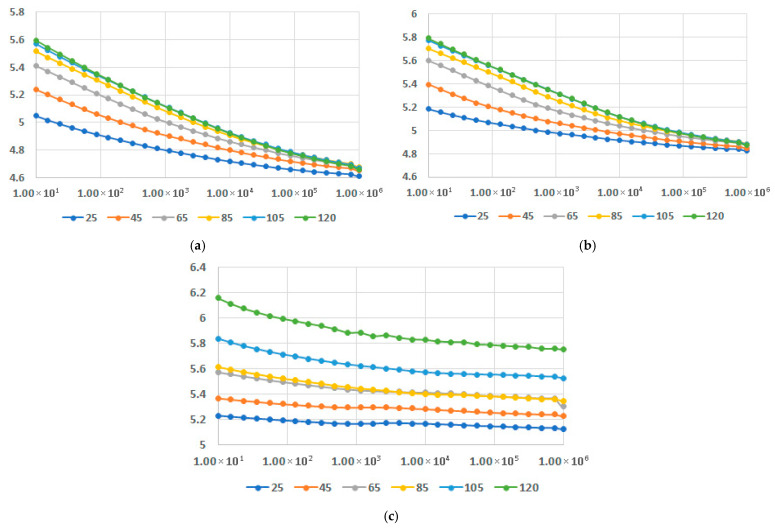
Dielectric permittivity vs. frequency [Hz] at different temperatures [°C] for (**a**) M7, (**b**) M8, and (**c**) M9.

In Figure 13, Figure 14 and Figure 15, dielectric permittivity at different temperatures is presented for M1-M9 samples. The temperature was selected to range between 25 °C and 120 °C, because the use of such equipment is for the interior of the vehicle, on one side, and on the other side, after all, and the thermoplastic material must correspond to the class E (120 °C) of insulation (temperature) with a maximum temperature rise till the hot spot margin of 120 °C and a recommended ambient temperature of 40 °C [61].

In all instances, it is obvious that the increase in fly ash content leads to increased values of permittivity. In regard to the variation in permittivity with temperature, for all contents of fly ash and for all matrices the values increased with the increase in temperature. It should be noted that it is a common phenomenon for the permittivity to gradually decrease as the frequency increases. The novelty is related to the fact that the decrease is lowered for a higher content of fly ash, as seen in Figure 13c and Figure 14c, and Figure 15c, and more divergent at higher frequencies. The case is even exceptional for PPDE, Figure 15c, where until 45 °C the characteristic is practically constant versus frequency, with a higher value of permittivity exceeding five.

**Figure 16:**
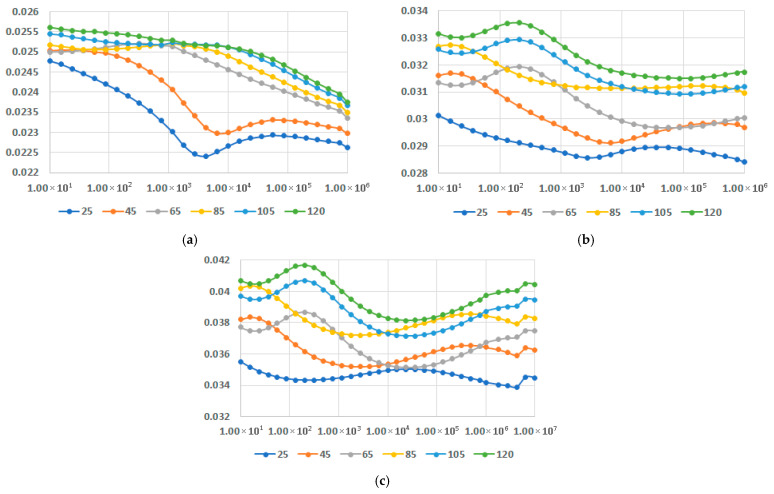
Dielectric loss factor vs. frequency [Hz] at different temperatures [°C] for (**a**) M1, (**b**) M2, and (**c**) M3.

**Figure 17:**
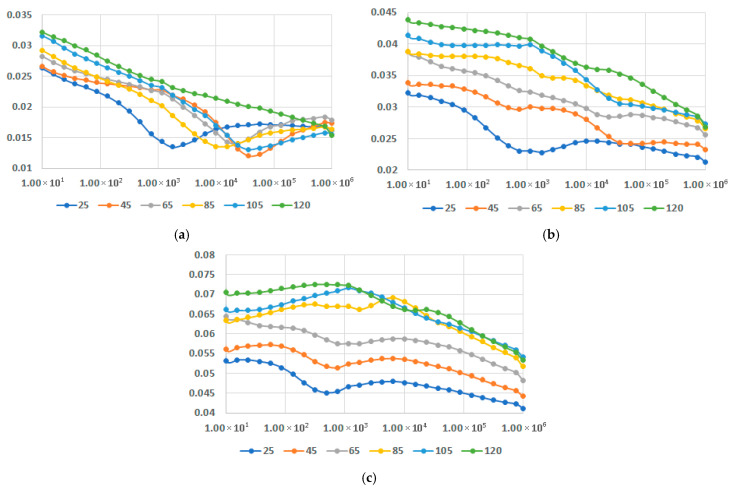
Dielectric loss factor vs. frequency [Hz] at different temperatures [°C] for (**a**) M4, (**b**) M5, and (**c**) M6.

**Figure 18:**
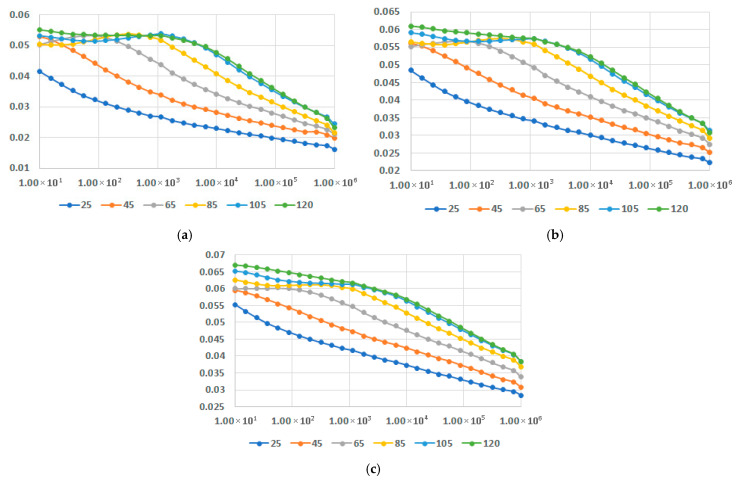
Dielectric loss factor vs. frequency [Hz] at different temperatures [°C] for (**a**) M7, (**b**) M8, and (**c**) M9.

In Figure 16, Figure 17 and Figure 18, dielectric loss factor at different temperatures is presented for M1-M9 samples. In all instances the interfacial polarization can be identified at lower frequencies followed by the dipolar polarization at frequencies around 10^4^–10^5^ Hz, depending on the recipe, where a maximum of the characteristics may be identified. When the temperatures rise, the maximum is translated progressively towards lower frequencies. The effect is more relevant for LDPEDE, e.g., Figure 16 c when compared to PPDE, Figure 18c. In all cases, the values of the dielectric loss factor increase with the content of fly ash and with the increase in temperature for all matrices. On the other hand, also for this characteristic, the novelty is related to the fact that the decrease is lowered for a higher content of fly ash, as seen in Figure 16c and Figure 17c, and Figure 18c, and more divergent at higher frequencies. The exceptional case is for LDPEDE, Figure 16c, where until 45 °C the characteristic is practically constant versus frequency. But the highest value of dielectric loss at higher frequencies is reached by HDPEDE composites, Figure 17c, with values exceeding 0.04.

**Figure 19:**
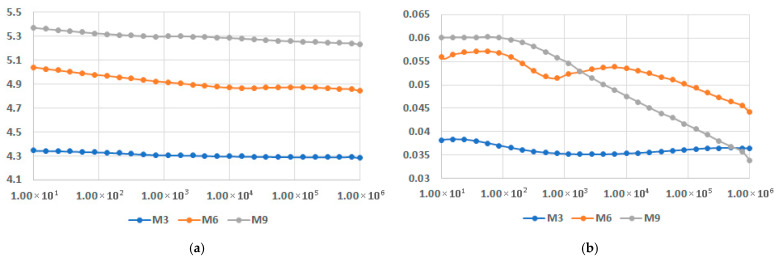
Dielectric permittivity (**a**) and dielectric loss factor (**b**) vs. frequency [Hz] for the highest fly ash content.

Figure 19 presents the comparison of dielectric permittivity and dielectric loss factor for the highest fly ash content, for the matrices involved, at 45 °C—the most common temperature of service for the derived components based on such composites. In all cases, the permittivity is practically invariant with the frequency. The highest value is reached by M9, which is of about 5.3, followed by M6, which is of about 4.9. In the case of dielectric loss factor, a slight variation with the frequency can be noticed for M3 and M6, but it is more significant in the case of M9. The most reliable characteristic is considered M6 in this instance, with values exceeding 0.045.

**Figure 20:**
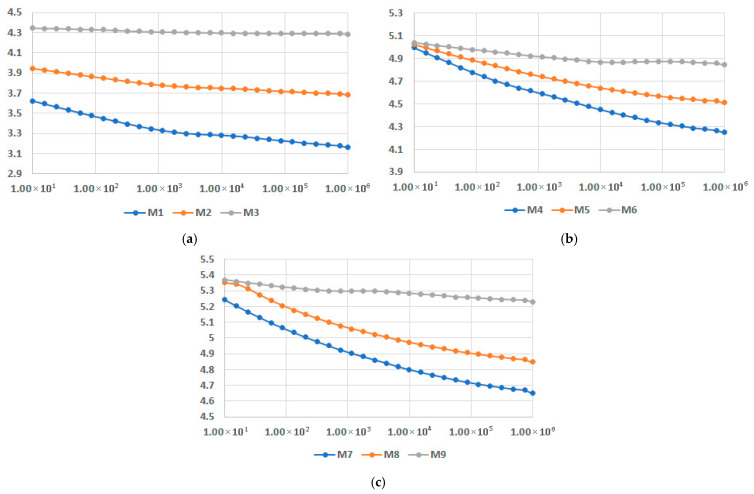
Dielectric permittivity vs. frequency [Hz] for different fly ash content: (**a**) LDPEDE, (**b**) HDPEDE, and (**c**) PPDE.

**Figure 21:**
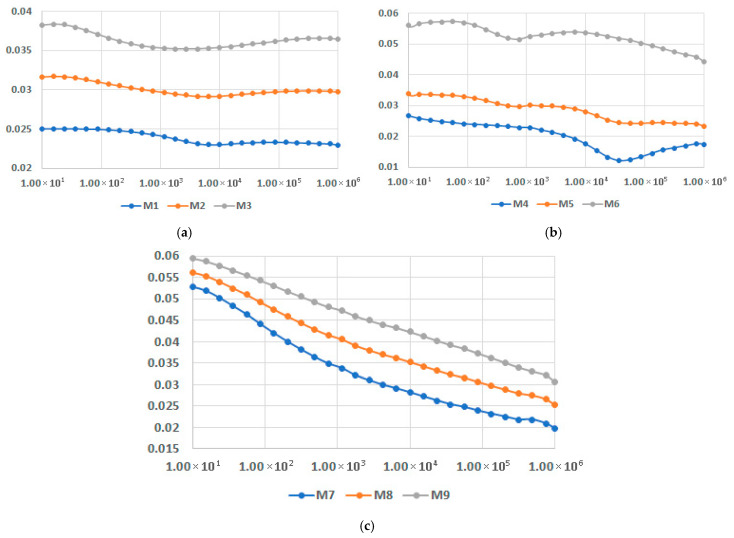
Dielectric loss factor vs. frequency [Hz] for different fly ash content: (**a**) LDPEDE, (**b**) HDPEDE, and (**c**) PPDE.

Finally, the comparison of dielectric permittivity and dielectric loss factor for variable fly ash content, for the matrices involved, at 45 °C, is presented in Figure 20 and Figure 21. It is confirmed that the characteristics substantially increase with the addition of fly ash. On the other hand, at the highest content of fly ash both dielectric permittivity and dielectric loss factor tend to become constant versus frequency, with only one exception: dielectric loss factor for PPDE, as shown in Figure 21c. According to this comparison, the recommended recipe might be M6, with the highest values of characteristics at higher frequencies.

Table 9 presents the electrical and electromagnetic features of composites.

In regard to the electrical features of composites, it was expected that the dielectric strength and resistivity would decrease with increased fly ash content due to the composition of fly ash, including semiconductive particles instead of talc which is an insulator. This observation is valid for all polymeric matrices. It is important to highlight that, when comparing the M3, M6 and M9 recipes, M6 continues to exhibit the highest values for dielectric strength and resistivity, a characteristic that supports the preliminary results regarding the dielectric features. Conversely, the significant reduction in surface and volume resistivity (even up to 10 times in the case of LDPEDE matrix) through the addition of fly ash may provide added characteristics associated with electric charge dissipation, a crucial requirement for electronic equipment in the automotive sector.

The research can be expanded by examining the impact of talc and fly ash, along with elastomer, in comparison to similar materials containing inorganic additives, as shown in [44], utilizing the same recycled polymer matrices. Even if the values of dielectric permittivity and loss factor are similar, due to the presence in both cases of 10% inorganic additive with similar features (in our case fly ash, and semiconducting powder in [44]) the shape of the characteristics are superior in our case, as presented in Figure 19 and Figure 21a,b, respectively, for both dielectric permittivity and dielectric loss factor as they tend to become constant versus frequency. This peculiar occurrence is a result of talc and elastomer presence, which join various forms of polarization with fly ash, balancing the general polarization effects across the frequency spectrum.

The effectiveness of electromagnetic interference shielding is presented in Table 9 as attenuation at two frequencies, specific for the usage of electronic equipment with IoT support. The attenuation depends on several factors, including the shielding material, its thickness, the signal frequency, and the shape of the enclosure. Taking into account the achieved data, M3, M6, and M9 may ensure a shielding performance of at least 99% (an attenuation of over −20 dB) at 2 mm thickness for the frequency interval of 0.1–4 GHz, a value which might be increased by an adequate design of the enclosures. The highest attenuation at both 0.1 and 4 GHz was achieved by M6 too.

#### 3.2.7. Thermal Shielding Evaluation

An important feature of electronic enclosures is related to their capacity to disperse the heat generated during the usage of electrical/electronic equipment. For this reason, the thermal shielding performance must be evaluated, as seen in Table 10. The polyethylene and polypropylene matrices are known as good thermal insulators, with a thermal conductivity of about 0.2 W/(m*K). On the other hand, talc itself is a good thermal insulator, possessing a thermal conductivity of about 0.15 W/(m*K). Therefore, the composites made solely of talc (M1, M4, M7) were expected to exhibit a thermal conductivity that is less than that of the corresponding polymer matrices. But due to the composition of fly ash, containing metal oxides with higher thermal conductivity, and to the composites structures, the composites with a higher percentage of fly ash can better transfer the heat and consequently their diffusivity and thermal conductivity values are higher. This observation is valid for all polymeric matrices, but it seems that the composites with PPDE (e.g., M9) exhibited higher values compared to HDPEDE (e.g., M6). Nevertheless, values of thermal conductivity of over 0.25 W/(m*K) are considered reasonable for electric car components, and practically there are samples at the highest content of fly ash in line with this requirement.

#### 3.2.8. Mechanical Properties

In contrast to traditional polymers, the mechanical characteristics of polymer composites can be customized by selecting the most adequate ingredient materials, their concentrations, along with the processing conditions used. The strength and stiffness of the fillers, which generally act synergistically with the polymer matrices, may significantly improve these properties. For this reason, talc was among the selected options concerning inorganic additives together with the fly ash, which could enhance the overall mechanical features. The results of the mechanical properties are briefly presented in Table 11. At a first view, according to expectations the addition of fly ash slightly diminished the values of the Shore hardness, elongation, and mechanical resistance in all circumstances related to the polymer matrix used. The elongation is higher in the case of LDPEDE, also due to the combined effect provided by the elastomer to the structure of the respective polymer. This is an aspect not met by the other polymers, which are more rigid, a factor already perceived when the Shore hardness was examined. In all, both M6 and M9 recipes present reasonable values for mechanical properties, but the shock resistance value of M6 is clearly superior, a factor required for automotive applications. This finally recommends M6 as the best recipe from the mechanical point of view.

The values achieved for the mechanical properties are in line with the technical requirements for basic electronic enclosures. The study can be enlarged by analyzing the effect of talc and fly ash, and, respectively, elastomer and comparing them with homolog materials with inorganic additives, as presented in [44], using the same recycled polymer matrices. In our case, the elongation presents higher values (up to 100 times), especially when comparing homolog materials with HDPEDE and PPDE matrices, in spite of the higher percentage of talc due to the addition of elastomer. In regard to the mechanical resistance, the respective values are at least 10% higher due to the presence of talc as an additive for all polymer matrices involved, and the influence of the elastomer is negligible. Finally, the flow resistance values are about 10% lower in our case when comparing to the materials described in [44] due to the presence of a higher amount of inorganic fillers.

#### 3.2.9. Analysis of the Degree of Water and Solvent Absorption

One of the weathering and chemical resistance tests for automotive applications involves the amount of water and solvent (toluene) absorbed by the composite materials. The experimental results are presented in Table 12. With a 95% confidence level, the degree of swelling was calculated as the average of five measurements performed on five different samples, removing out-of-range values. It is already known that composites with inorganic powders absorb a higher amount of water and solvent (toluene) compared to the homolog polymer matrix only [26,44,62]. But this study expanded the experiments when fly ash was used as an ingredient. It is obvious that the absorption properties depend both on composite structure—given by the structure of the polymer matrix used—and on the type and concentration of the inorganic additive. In our case, LDPEDE determines a more porous structure, compared to HDPEDE and PP, which are denser. This aspect partially explains the much higher values of absorbed amounts of water and solvent (toluene) in the case of LDPEDE matrix. On the other hand, due to its composition fly ash presents metallic oxides with a higher affinity to water when partially substituting talc, which is less receptive; hence one can notice a significant difference between the values of, e.g., M3 vs. M1, M6 vs. M4, or M9 vs. M7. In regard to the solvent absorption, the differences are not so high when comparing recipes with different ash content, because in this case only the polymer structure and formula matter. But the absorption of solvent is clearly more significant when compared with the absorption of water, an aspect fully explainable when dealing with organic substances. In all, both M6 and M9 recipes present reasonable values for the amount of water and solvent (toluene) absorbed.

#### 3.2.10. Martens Thermal Stability

The thermal stability data are presented in Table 13.

The conclusions of the Martens thermal stability test are:

- Martens thermal stability [°C]: 90–110.
- Flammability class without flame retardant: V-2.
- Permanent service temperature [°C]: <80.
- Short-term high temperature resistance [°C]: 170–200.

These data are fully compatible with the best usage for electric car components and under the conditions imposed by the insulation class (temperature).

Taking into account all results presented above, we can appreciate that the recipe with 10% talc, 10% fly ash, and elastomer embedded within HDPEDE matrix fulfills all requirements for the use for electric car components, representing the best choice for such applications, eventually followed by the same composition embedded in PPDE.

The technical results are comparable with the features of homologous composites for electronic enclosures with a low percentage of conductive fillers, as presented in [63,64,65], but in those cases the composites are more complex, more expensive, and harder to recycle. In our situation, the benefits consist of a simpler technology, the utilization of recycled materials, being eco-friendly, and a very low cost of materials. Beyond being lightweight and offering good resistance to impact, chemicals, corrosion, and a pre-defined electromagnetic shielding, the actual problem related to plastic enclosures for automotive applications consists of the need to be recyclable in the context of the circular economy [66].

A preliminary view upon the recyclability of the materials developed by this research may be found according to the assumptions in [43] and by a cradle-to-gate analysis. The advantage of the materials developed by the presented research is that they are self-recyclable by the same thermoplastic technology, i.e., they can be used directly as raw material for similar components with a minimal addition of ingredients, especially specific agents for polymer regeneration and also compatibilizing and coupling agents, in order to achieve the optimal products.

## 4. Conclusions

In this study, thermoplastic waste (polyethylene and propylene) from WEEE was used for producing polymer composite materials incorporating talc, fly ash, and elastomers intended for electric car components with customized electromagnetic interference shielding features. The compositions were balanced from 20% talc to 10% talc and 10% fly ash, with all recipes including 10% elastomer. The analysis upon the fly ash revealed a wide range of metallic oxides, some of which are compatible with talc and improve talc’s positive impact upon mechanical properties, while others provide potential ferritic characteristics needed for an efficient electromagnetic interference shielding.

A relatively uniform dispersion of inorganic ingredients without particle agglomeration was noticed, facilitated by the elastomer, indicating an efficient mixing during melt compounding.

Although the percentage of inorganic components in composites remains 20%, the progressive substitution of talc with fly ash reduces the fluidity index and softening temperature values. A possible explanation when also considering the reduction in both bulk and melt density values may be based on the lower density of fly ash in comparison to talc powder.

The increase in fly ash content leads to increased values of both permittivity and dielectric loss factor. Concerning the change in permittivity and dielectric loss with temperature, the values substantially increased with increasing temperature for all fly ash contents and all polymer matrices. The novelty pertains to the observation that at higher amounts of fly ash, more divergent characteristics occurred for both characteristics at higher frequencies, tending to a constant value. Very relevant values were reached for the permittivity, exceeding five, and for dielectric loss factor, exceeding 0.04, a prerequisite for their use for electrical equipment purposes.

The dielectric strength and resistivity values slightly decreased with increased fly ash content, but the respective values remained functional.

The recipes with 10% fly ash may assure an electromagnetic shielding efficiency of no less than 99% (an attenuation exceeding −20 dB) at a thickness of 2 mm for the frequency range of 0.1–4 GHz, values that could be enhanced through a more professional design of the enclosures.

Composites containing a higher content of fly ash can transfer heat more effectively, resulting in increased values for diffusivity and thermal conductivity, with very relevant values being reached for the thermal conductivity as they exceed 0.2 W/(m*K), in line with the requirements for electric vehicle parts applications.

The analysis of mechanical characteristics, of degree of water and solvent absorption, and of thermal stability confirmed that the composites with high values of fly ash present functional values in line with the applications for automotive industry.

Considering all the results mentioned above, we can estimate that the recipes containing 10% talc, 10% fly ash, and elastomer compounded within HDPEDE, followed by PPDE, may represent the optimal selection for electric car components as they meet all necessary criteria for such applications.

## Figures and Tables

**Table 1:** Composition and labeling of the studied materials.

Samples Labeling	Matrix	Component Concentration	
		Ta (wt.%)	FA (wt.%)
M1	LDPEDE HDPEDE	20	0
M2		15	5
M3		10	10
M4		20	0
M5		15	5
M6	PPDE	10	10
M7		20	0
M8		15	5
M9		10	10

**Table 2:** Melting intervals for the obtained composites.

Melting Interval (°C)				
**M1**	**M2**	**M3**	**M4**	**M5**
115–130	115–135	120–135	130–150	135–155
**M6**	**M7**	**M8**	**M9**	
135–155	155–170	160–170	160–175	

**Table 3:** XRF analysis of fly ash.

Formula	Z	Concentration	Line	Net Int.	Stat. Error	Analyzed Layer
SiO_2_	14	32.53%	Si KA1-HR-Tr	29.94	1.03%	4.4 μm
CaO	20	21.19%	Ca KA1-HR-Tr	90.50	0.595%	19.3 μm
Al_2_O_3_	13	10.15%	Al KA1-HR-Tr	10.44	1.77%	4.0 μm
Fe_2_O_3_	26	8.13%	Fe KA1-HR-Tr	145.3	0.471%	48 μm
Br	35	7.35%	Br KA1-HR-Tr	595.6	0.233%	195 μm
CuO	29	3.82%	Cu KA1-HR-Tr	33.97	0.974%	74 μm
PbO	82	2.73%	Pb LB1-HR-Tr	82.75	0.653%	228 μm
TiO_2_	22	2.45%	Ti KA1-HR-Tr	10.02	1.80%	20.7 μm
SnO_2_	50	2.38%	Sn KA1-HR-Tr	34.54	1.13%	0.95 mm
BaO	56	2.13%	Ba LA1-HR-Tr	3.999	2.88%	20.3 μm
P_2_O_5_	15	1.26%	P KA1-HR-Tr	1.570	4.65%	4.6 μm
SrO	38	1.08%	Sr KA1-HR-Tr	90.00	0.626%	215 μm
MgO	12	0.95%	Mg KA1-HR-Tr	0.7436	8.03%	2.66 μm
K_2_O	19	0.81%	K KA1-HR-Tr	3.766	2.97%	14.9 μm
SO_3_	16	0.75%	S KA1-HR-Tr	1.781	4.43%	6.3 μm
ZrO_2_	40	0.66%	Zr KA1-HR-Tr	69.03	0.729%	278 μm
Cl	17	0.54%	Cl KA1-HR-Tr	1.985	4.26%	8.2 μm
ZnO	30	0.36%	Zn KA1-HR-Tr	19.26	1.40%	90 μm
Cr_2_O_3_	24	0.28%	Cr KA1-HR-Tr	2.624	3.89%	31 μm
MnO	25	0.10%	Mn KA1-HR-Tr	1.587	5.61%	39 μm

**Table 4:** XRF analysis of LDPEDE.

Formula	Z	Concentration	Line 1	Net Int.	Stat. Error	LLD	Analyzed Layer
C (organic)	6	98.4%	Matrix				
TiO_2_	22	0.67%	Ti KA1-HR-Tr	43.19	0.866%	16.2 PPM	206 μm
CaO	20	0.62%	Ca KA1-HR-Tr	30.93	1.02%	13.8 PPM	124 μm
Cl	17	0.10%	Cl KA1-HR-Tr	4.007	2.94%	19.3 PPM	45 μm
SiO_2_	14	0.09%	Si KA1-HR-Tr	0.7221	6.66%		13.4 μm
P_2_O_5_	15	0.05%	P KA1-HR-Tr	0.6496	7.52%	29.8 PPM	20.5 μm
ZnO	30	0.01%	Zn KA1-HR-Tr	16.54	1.82%	4.3 PPM	1.32 mm
Fe_2_O_3_	26	0.01%	Fe KA1-HR-Tr	4.522	3.47%	7.7 PPM	0.53 mm

**Table 5:** XRF analysis of HDPEDE.

Formula	Z	Concentration	Line 1	Net Int.	Stat. Error	LLD	Analyzed Layer
C (organic)	6	93.7%	Matrix				
CaO	20	4.84%	Ca KA1-HR-Tr	192.8	0.408%	20.5 PPM	114 μm
TiO_2_	22	0.46%	Ti KA1-HR-Tr	16.66	1.40%	23.5 PPM	117 μm
SiO_2_	14	0.31%	Si KA1-HR-Tr	2.346	3.77%	51.1 PPM	12.9 μm
Cl	17	0.21%	Cl KA1-HR-Tr	7.331	2.15%	23.3 PPM	42 μm
MgO	12	0.09%	Mg KA1-HR-Tr	0.5441	8.51%	70.5 PPM	5.1 μm
Fe_2_O_3_	26	0.07%	Fe KA1-HR-Tr	12.70	1.69%	10.2 PPM	0.30 mm
CuO	29	0.02%	Cu KA1-HR-Tr	1.533	4.71%	4.4 PPM	0.58 mm
ZnO	30	0.01%	Zn KA1-HR-Tr	7.896	2.74%	6.0 PPM	0.71 mm
Br	35	48 PPM	Br KA1-HR-Tr	5.695	4.99%	5.1 PPM	1.82 mm

**Table 6:** XRF analysis of PPDE.

Formula	Z	Concentration	Line 1	Net Int.	Stat. Error	LLD	Analyzed Layer
C (organic)	6	92.2%	Matrix				
CaO	20	5.90%	Ca KA1-HR-Tr	221.0	0.381%	21.5 PPM	110 μm
TiO_2_	22	0.37%	Ti KA1-HR-Tr	12.02	1.65%	24.3 PPM	106 μm
SiO_2_	14	0.36%	Si KA1-HR-Tr	2.649	3.48%		12.7 μm
Cl	17	0.23%	Cl KA1-HR-Tr	7.558	2.11%	22.7 PPM	41 μm
MgO	12	0.11%	Mg KA1-HR-Tr	0.6985	7.30%	67.7 PPM	5.1 μm
P_2_O_5_	15	0.08%	P KA1-HR-Tr	0.8862	6.33%	33.8 PPM	19.1 μm
Fe_2_O_3_	26	0.06%	Fe KA1-HR-Tr	8.673	2.10%	11.2 PPM	275 μm
SO_3_	16	0.05%	S KA1-HR-Tr	0.9963	6.24%	27.4 PPM	28.3 μm
ZnO	30	0.01%	Zn KA1-HR-Tr	7.457	2.80%	6.4 PPM	0.64 mm
CuO	29	93 PPM	Cu KA1-HR-Tr	0.7932	6.73%	4.9 PPM	0.52 mm
Br	35	45 PPM	Br KA1-HR-Tr	4.833	5.70%	5.6 PPM	1.65 mm
SrO	38	28 PPM	Sr KA1-HR-Tr	4.145	7.55%	4.9 PPM	2.74 mm

**Table 7:** Experimental results regarding the vitreous transitions for recycled matrices.

Sample	Melting	Vitreous Transitions		
	Specific Heat [J/g]	Specific Heat Capacity [J/g*K]	Initial Temperature [°C]	Final Temperature [°C]
LDPEDE	102	0.72	203.9	234.6
HDPEDE	128.4	1.128	242.7	252.9
PPDE	77.41	-	-	-

**Table 8:** Physical analysis, error < 1% [48,49,50].

	Unit	Samples								
Characteristic		M1	M2	M3	M4	M5	M6	M7	M8	M9
Fluidity index	g/10 min	0.95	0.92	0.90	0.63	0.62	0.60	1.123	1.089	1.067
Melt density	kg/m^3^	781	776	769	754	749	744	841	839	832
Bulk density	g/cm^3^	0.919	0.915	0.911	0.928	0.923	0.919	0.931	0.926	0.922
Ash content	%	21.38	21.13	21.08	21.01	20.79	20.52	20.62	20.43	20.25
Softening temperature VICAT	°C	118	117	113	137	136	133	143	142	140

**Table 9:** Electrical and electromagnetic features, error < 1% [55,56,57].

	Unit	Samples								
Characteristic		M1	M2	M3	M4	M5	M6	M7	M8	M9
Dielectric strength	kV/mm	53.5	52.8	52.5	54.8	54.5	54.1	54.4	53.7	52.9
Surface resistivity	Ω	1.4 × 10^9^	5.7 × 10^8^	3.3 × 10^8^	3.5 × 10^9^	2.6 × 10^9^	1.3 × 10^9^	4.1 × 10^9^	2.8 × 10^9^	1.9 × 10^9^
Volume resistivity	Ω cm	2.1 × 10^9^	7.8 × 10^8^	5.2 × 10^8^	5.4 × 10^9^	3.5 × 10^9^	2.6 × 10^9^	4.8 × 10^9^	3.8 × 10^9^	2.3 × 10^9^
Attenuation 0.1 GHz	-dB	13.62	21.61	24.27	13.55	22.40	25.36	13.65	22.14	24.98
Attenuation 4 GHz	-dB	18.75	25.04	28.44	18.64	26.85	32.67	18.52	25.96	31.12

**Table 10:** Thermal shielding performance, error < 1%.

	Unit	Samples								
Characteristic		M1	M2	M3	M4	M5	M6	M7	M8	M9
Diffusivity	mm^2^/s	0.198	0.224	0.229	0.199	0.222	0.248	0.224	0.236	0.269
Thermal conductivity	W/(m*K)	0.179	0.193	0.217	0.181	0.189	0.227	0.207	0.223	0.251
Specific heat capacity	J/g/K	2.547	2.319	2.272	2.726	2.545	2.334	2.397	2.246	2.029

**Table 11:** Mechanical properties, error < 1% [52,54].

	Unit	Samples								
Characteristic		M1	M2	M3	M4	M5	M6	M7	M8	M9
Shore hardness Shore D	^0^Sh D	46	44	40	61	60	56	68	67	62
Shock resistance CHARPY	KJ/m^2^	-	-	-	22.6	22.4	22.1	8.6	8.4	8.2
Mechanical resistance	MPa	13.92	13.55	13.25	16.88	16.27	16.11	19.46	18.84	18.45
Flow resistance	MPa	2.62	1.84	1.37	6.25	6.14	5.84	7.05	6.84	6.38
Elongation	%	188	159	132	27	24	18	22	19	16
Young’s Modulus	GPa	0.25	0.19	0.14	0.58	0.57	0.56	0.62	0.59	0.57

**Table 12:** Water and solvent absorption data, error < 1% [58].

	Unit	Samples								
Characteristic		M1	M2	M3	M4	M5	M6	M7	M8	M9
Water	%	0.25	0.38	0.96	0.18	0.36	0.67	0.16	0.31	0.62
Solvent	%	2.13	2.20	2.28	1.86	2.04	2.17	1.83	2.01	2.16

**Table 13:** Thermal stability data, error < 0.1% [60].

	Unit	Samples								
Characteristic		M1	M2	M3	M4	M5	M6	M7	M8	M9
Thermal stability	°C	94	92	90	96	94	92	95	93	91

## Data Availability

The data are included within the paper.
